# Optimisation of Bioinspired Fibre Architectures for 3D-Printed Polymer Heart Valves via Melt Electrowriting (MEW) Using FE Modelling and Design of Experiments (FE-DOE)

**DOI:** 10.3390/biomimetics11060421

**Published:** 2026-06-13

**Authors:** Celia Hughes, Robert D. Johnston, Dylan Armfield, Desmond McCarthy, Ewa Klusak, Emily Growney, Evelyn Campbell, Caitríona Lally

**Affiliations:** 1 Trinity Centre for Biomedical Engineering, Trinity Biomedical Sciences Institute, Trinity College Dublin, D02 PN40 Dublin, Ireland; cehughes@tcd.ie (C.H.); robertjohnston@rcsi.ie (R.D.J.); mccard18@tcd.ie (D.M.); 2 Department of Mechanical, Manufacturing, and Biomedical Engineering, School of Engineering, Trinity College Dublin, D02 PN40 Dublin, Ireland; 3 Structural Heart Division, Boston Scientific Corporation, H91 Y868 Galway, Ireland; ewa.klusak@bsci.com (E.K.); emily.growney@bsci.com (E.G.); evelyn.campbell@bsci.com (E.C.); 4 Advanced Material and Bioengineering Research Centre (AMBER), Trinity College Dublin, D02 PN40 Dublin, Ireland

**Keywords:** aortic valve, TAVI, polymer valve, melt electrowriting, finite element modelling, Design of Experiments

## Abstract

Aortic stenosis is predominantly treated through transcatheter bioprosthetic heart valve implantation. However, the materials used in these devices are prone to premature failure. Polymer heart valves provide an alternative to current commercial devices, offering materials with greater durability and customisation through fibre reinforcement. Given the wide range of available materials and structures, there is a need for a systematic and efficient approach to designing and optimising novel bioinspired polymeric leaflets. This work presents a framework that employs computational modelling and Design of Experiments (DOE) tools to optimise bioinspired, 3D-printed, fibre-reinforced polymer leaflets made using melt electrowriting (MEW). Here, finite element (FE) models are created to represent MEW fibre-reinforced polymer leaflets for application in a transcatheter aortic heart valve. The behaviour of this valve under physiological loading conditions is modelled to predict valve performance and leaflet material response. These models were first used to investigate the impact of fibre orientation on valve performance and leaflet response, thereby demonstrating the benefits of a bioinspired fibre reinforcement structure. Using a DOE approach, the structural combination of MEW fibre reinforcement and an elastomeric matrix was optimised to improve valve performance and reduce leaflet stress and strain. Overall, the framework offers an efficient and versatile methodology for optimising fibre-reinforced polymer leaflets using an in silico approach, thereby reducing the need for physical prototyping and testing of these next-generation devices during early product development.

## 1. Introduction

Aortic stenosis is a prevalent disease of the aortic valve, affecting 4% of people over the age of 70 in the US, and leads to a 50% chance of mortality within two years if left untreated [1]. When surgical methods are not indicated, most treatment is delivered via minimally invasive approaches, such as transcatheter aortic valve implantation (TAVI) [2]. In this case, the implanted valves contain leaflets made from biological materials, either porcine or bovine pericardium, and may experience premature failure due to material failure and/or calcification [3]. The pericardium is a stiff, collagenous tissue which can potentially improve the durability of TAVI devices if the collagen fibres within the pericardial leaflets are aligned to bear the load [4]. However, robust fibre screening methods are not currently utilised or required in the manufacture of these devices [5], and it would be challenging to source tissue with an idealised fibre structure for leaflet load-bearing.

Polymer heart valves offer the potential to provide a durable, biocompatible alternative to currently available commercial devices. A wide variety of polymer heart valves are currently under development, employing innovative approaches to create these next-generation devices. These studies have primarily focused on developing bespoke polymer blends in order to achieve superior material durability and mechanical response compared to silicone or rubber. Examples of these valves are the Tria valve [6,7], PolyNova valve [8,9], PoliValve [10], HA-LLDPE valve [11], and Life Polymer [12]. Notably, the Tria valve has recently become the first ever commercially available polymer heart valve approved for use, gaining approval in India for use in the mitral position [13].

Some polymer valve development has also focused on integrating a customisable fibre reinforcement structure. This can be controlled using novel polymers, such as the copolymer blocks used in the PoliValve, which can be arranged to give a mechanically relevant orientation [10]. Fibres can also be applied by leaflet additions, such as the 3D-printed valve developed by Coulter et al. [14], which has bioinspired fibres based on the native aortic valve fibre structure overlaid on a polymer base [14,15]. Commissural and free-edge fibres are also added in new generations of an HA-LLDPE valve [11]. Additionally, embedded leaflet reinforcements have been explored using nitinol fibres to support leaflet stresses in the radial direction [16] and integration of a nylon fibre mesh [17]. Many of these studies have shown reductions in leaflet strains and improved hydrodynamic performance with these fibrous inclusions [11,14,16,18].

In addition, there has been considerable development in bioinspired, tissue-engineered valve leaflets [19,20,21,22]. While these devices still require many years of development before they can be made commercially available, they have shown potential for adopting manufacturing methods such as melt electrowriting (MEW) for the creation of bioinspired fibre reinforcement structures [21]. In this case, “bioinspired” refers to the anisotropic stiffness of the leaflet, induced by the fibre structure, paired with fibre crimping to achieve the familiar J-shaped curve seen in soft biological tissues. MEW is a highly controllable extrusion-based 3D printing technique that can produce structures with fibres at a biologically relevant scale [23]. MEW offers advantages over traditional FDM approaches in that it is able to produce highly controllable structures with fibre diameters ranging from >20 µm to <1 µm [24].

Previous work in our lab has studied the mechanical response and collagen microstructure of native porcine aortic valve leaflets [25]. Using this as a baseline, MEW can be used to manufacture a bioinspired fibre reinforcement structure, emulating the orientation of the native leaflet collagen fibre structure, which will be embedded in a compliant elastomeric matrix to mimic the structure and mechanical response of aortic valve leaflets.

Finite element modelling is a powerful and versatile tool that can aid the design and development of polymer valves. It has been used primarily to improve existing designs by modifying leaflet thickness or shape, or by adding simple reinforcement structures [11,14,16,25,26], with the potential to transform the design, development, and assessment processes of these devices. By leveraging FE modelling, we can assess novel material combinations and structures in silico to guide future manufacturing and bench testing. Implementation of these tools would dramatically decrease development time for these devices by reducing the manufacturing and assessment time of potential material combinations.

Design of Experiments (DOE) is another valuable tool for developing novel valve leaflets. With a seemingly endless range of materials and structural features to choose from when designing composite, bioinspired leaflets, DOE can aid the design process. By providing a systematic approach to efficiently test combinations of features, DOE can enable sound statistical analysis of each feature’s impact [26]. DOE has been used alongside FE modelling to improve manufacturing workflows and optimisation for applications such as rotary endodontic instruments [27], circular saw tooth geometry [28], and steel bridge stiffeners for bridge assembly [29].

This study develops an in silico FE and DOE framework to design and evaluate fibre-reinforced polymeric heart valve leaflets for transcatheter applications. MEW-enabled fibre architectures are parameterised within a compliant elastomeric matrix to represent additively manufacturable composite leaflets, and their structural performance is assessed under physiological loading conditions. By systematically varying fibre orientation and architectural features, the framework establishes quantitative relationships between printable design parameters and valve-level performance metrics. The objective is to use this computational workflow to identify optimised MEW-manufacturable reinforcement structures and to assess the mechanical advantages of bioinspired fibre orientations.

## 2. Materials and Methods

To simulate a fibre-reinforced leaflet, a model with discrete fibre inclusions was initially developed to obtain the mechanical response of a MEW–elastomer composite. This response was used to calibrate a hyperelastic, fibre-reinforced constitutive model to describe the material response and fibre orientations in a trileaflet valve model. This methodology was first used to verify the benefit of bioinspired fibre orientations. It was then used alongside a DOE approach to determine the optimal MEW and elastomer structural combination.

In this work, the simulated material was Sylgard-184 (Dow Inc., Midland, MI, USA) polydimethylsiloxane (PDMS) at a 16:1 base-to-curing volume ratio, embedded with a polyether ether ketone (PEEK)-like fibre structure.

### 2.1. Discrete Fibre Model

**Figure 1:**
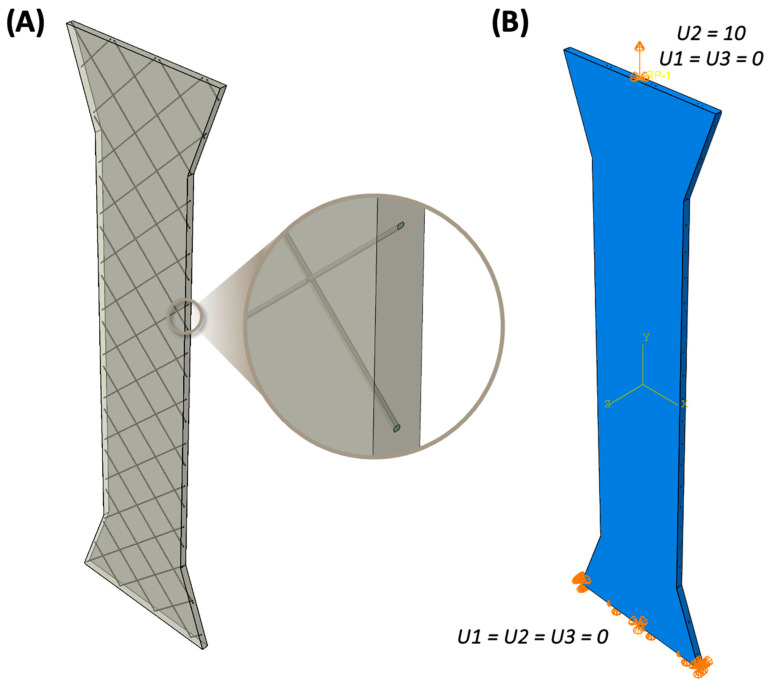
Uniaxial dogbone model with discrete fibre inclusions showing (**A**) fibres embedded in matrix with different material assignments, and (**B**) model boundary conditions.

A dogbone-shaped model with discrete fibre inclusions was created in Abaqus 2022 (SIMULIA, Dassault Systèmes, Paris, France) to simulate the mechanical response of a MEW-embedded elastomer. To create this, a dogbone model and a MEW mesh were created separately. The dogbone shape had a gauge length of 18 mm, a width of 4.5 mm, and variable thickness. A MEW mesh was created in SolidWorks (SolidWorks Corp., Dassault Systèmes, 2023, Paris, France) with an assumed circular fibre diameter of 20 µm. The mesh cross-section was created using a slot shape, with a thickness of 20 µm and a height of 20 µm multiplied by the total number of layers. Layer number and fibre spacing were variable. All layers were modelled as a solid part, see Figure 1A.

In Abaqus CAE, the two parts were merged together with the boundaries retained. Each component was assigned unique material properties and meshed as a single solid part. For mesh generation, the part was seeded with an element size of 0.0625 mm for the fibres and 0.2 mm on the outside edges. It was meshed with 4-node linear tetrahedron elements. The matrix component, PDMS, was modelled as a first-order Ogden material with a µ_1_ = 0.204 MPa, α_1_ = 2.504, and D = 0; the material parameters were calibrated from previous in-house uniaxial tensile testing using Abaqus (see Supplementary Materials, Figure S1). The Ogden hyperelastic model is commonly used to describe PDMS mechanical behaviour [30,31]. A mesh sensitivity analysis and material calibration curves are available in the Supplementary Materials, Figure S2. The fibres were modelled as a linear elastic material based on PEEK with a Young’s modulus of 2906 MPa and a Poisson’s ratio of 0.45 [32]. To simulate a uniaxial test, the bottom surface of the dogbone was fixed, and the top surface was constrained using kinematic coupling to a reference point; see Figure 1B. A displacement boundary condition of 10 mm was applied to the reference point to deform the model.

The true stress–strain response of this material was calculated from the force–displacement output recorded at the reference point. The built-in Gasser–Ogden–Holzapfel (GOH) model in Abaqus [33] was calibrated using Mcalibrate (PolymerFEM, Needham, MA, USA), where the material parameters were identified through least-squares minimisation between the stress–strain response and the model-predicted response. Testing of the GOH model on these structures is presented in the Supplementary Materials, Figures S6–S8.

### 2.2. Trileaflet Valve Model

A trileaflet valve model inspired by the ACURATE neo2 Aortic Valve™ (Boston Scientific, Marlborough, MN, USA) was developed to simulate the behaviour of MEW-embedded polymer leaflets in a simulated valve environment.

#### 2.2.1. Geometry, Mesh, Material, and Fibre Orientations

**Figure 2:**
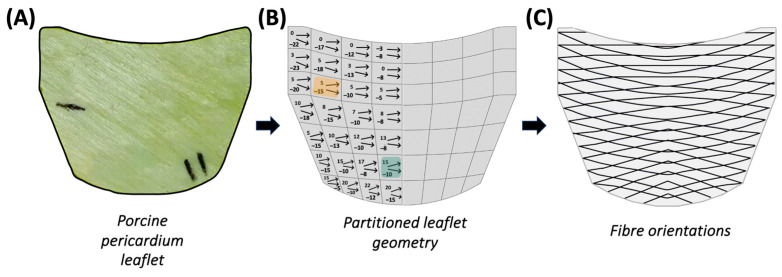
(**A**) Porcine pericardium leaflet from ACURATE neo2 aortic valve compared to (**B**) leaflet model including partitions and local fibre orientations, and (**C**) diagram of fibre orientations across leaflet. Orange and blue regions in (**B**) correspond to fibre orientations informed from imaging of native leaflets’ commissures and belly regions, respectively [24].

For the valve model, the leaflet geometry was designed based on the ACURATE neo2 Aortic Valve™. The leaflet geometry was partitioned into 56 discrete rectangular regions to assign local fibre orientations, and the regions were meshed using 22,715 full-integration C3D8 brick elements using ANSA Beta (Beta CAE Systems, Lucerne, Switzerland). Mesh convergence studies were performed and are documented in the Supplementary Materials, Figure S3. Each of the 56 regions was assigned a unique local fibre orientation for the two fibre families, as shown in Figure 2. These orientations were informed by SHG imaging-based measurements obtained in previously published results [25]; orientations for the belly region were assigned to a partition in the same region on the leaflet model, and the commissure orientations were assigned to a partition in the corresponding region. Fibre orientations were extrapolated based on these two regions for the rest of the leaflet, ensuring no family orientation differed by more than 5° from its neighbouring regions. Material behaviour in the leaflet was described using the GOH model, with parameters calibrated from the discrete fibre uniaxial model.

**Figure 3:**
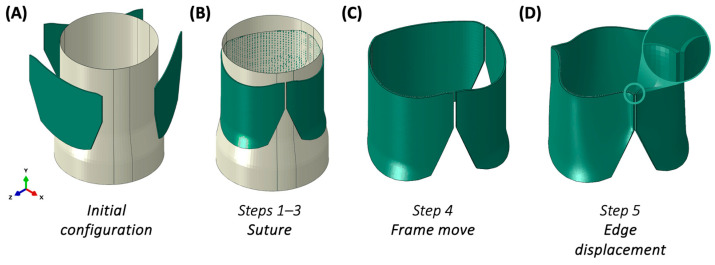
Valve model setup steps before pressure application to simulate sutured leaflet. (**A**) The initial configuration, (**B**) leaflet suturing to the frame, (**C**) moving the frame away from the leaflets, and (**D**) displacement of the leaflet edges.

To represent a reasonable approximation of leaflet suturing locations relative to the stent frame, an artificial surface geometry of the frame was included; see Figure 3A. This was meshed with 2608 SFM3D4R elements.

#### 2.2.2. Boundary Conditions and Steps

Pulsatile loading of the polymer valve was simulated using ABAQUS/Explicit. Eight sub-steps were used for the complete simulation: four steps to adjust the planar leaflets to their respective suture locations on the stent, one to remove the artificial frame from the simulation domain, and three pulsatile pressure-load steps; see Figure 3.

During steps 1, 2, and 3, the leaflet is brought into contact with the frame surface, its edges are aligned near the frame surface, and it is then pressed against the frame, respectively. Throughout these three steps, the frame was fixed in place. For step 3, a nominally low pressure (*p* = 0.005 MPa) was applied to the leaflet outer surface until the leaflets were fully in contact with the frame surface. The frame surface was no longer needed once the leaflets were correctly positioned and was removed from the domain; see Figure 3C.

To complete suturing, the inner edges of the leaflets were adjusted to contact one another as they would in a fully assembled valve; see Figure 3D. For a detailed view of boundary conditions applied throughout the model steps, see the Supplementary Materials, Figure S4.

**Figure 4:**
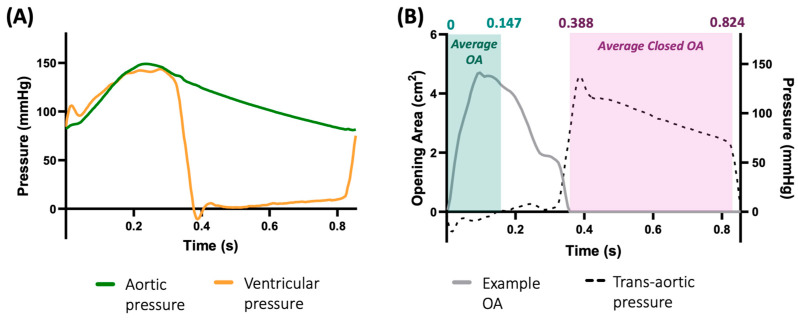
(**A**) The pressure applied to the aortic and ventricular surfaces across a step, and (**B**) a diagram showing resultant aortic pressure alongside an example opening area (OA) curve detailing the regions taken for average OA and average closed OA calculations.

Pressure was applied as a dynamic load to both the aortic and ventricular leaflet surfaces, as shown in Figure 4A, to simulate the pulsatile loading of the valve during a single cardiac cycle [5]. These curves, provided by Boston Scientific, were extracted from pulse duplicator testing on ACURATE neo2 devices that were tested using a ViVitro Pulse Duplicator system (ViVitro Labs Inc., Victoria, BC, Canada) under hypertensive pressure (peak trans-aortic pressure of 140 mmHg) and nominal flow conditions (70 bpm, 35% systolic duration, and 5 L/min), as outlined in ISO 5840 [34]. One full cycle, lasting 0.857 s, was applied across a single step. To minimise the effect of the initial geometry and any inertial effects from the previous step, the pressure conditions were repeated three times across steps 6, 7, and 8, and results were taken from the final step.

Contact for the leaflet–stent and leaflet–leaflet interactions was defined using the ABAQUS/Explicit general contact algorithm. The same interaction property was assigned to all contact pairs: tangential behaviour was formulated using the penalty algorithm with a friction coefficient of 0.1 [35]. Normal behaviour was defined using the default Hard contact algorithm with the default constraint enforcement method. Separation was allowed after contact.

#### 2.2.3. Valve Opening Area Extraction

**Figure 5:**
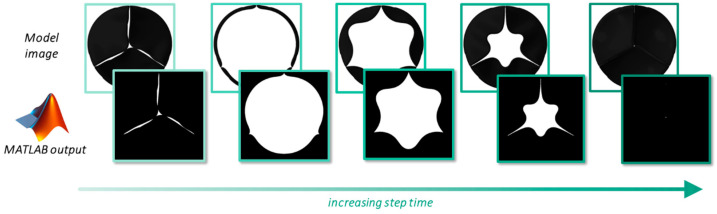
A diagram comparing the image of the valve from the top view extracted from Abaqus to the output from a custom MATLAB script to determine valve opening area.

Images of the valve’s top view were automatically extracted during the final loading cycle, totalling 128 images, each representing a time step of 0.0067 s. These were analysed in a custom MATLAB (MathWorks Inc. (2024), Natick, MA, USA) script that used edge detection to calculate the opening area of the valve; see Figure 5. With these outputs, a variety of parameters were extracted; see Figure 4B. First, the maximum opening area (OA) across the entire cycle was identified. However, since this is a single measurement from a period with varying areas, a secondary average OA was calculated as a surrogate for the effective orifice area (EOA). EOA is a clinical metric of valve opening performance derived from flow measurements, and for valves of the size considered, it is required to be at least 1.7 cm^2^ [34]. Average OA was defined as the mean area when ventricular pressure exceeded aortic pressure, which corresponded to 0–0.147 s. This aligns with methods employed by our commercial partners, in which EOA during pulse duplicator testing is determined from cardiac output in this region. This measurement was validated by modelling porcine pericardium leaflets and comparing the average OA measurement with expected EOA values; the results were in good agreement (see Supplementary Materials). A measure of closure, defined as the average OA at the end of the cycle (0.388–0.824 s), was also extracted. Finally, as a measure of valve “efficiency”, the time from the start of the cycle to maximum opening area was identified.

### 2.3. Fibre Orientation Verification

To determine the impact of bioinspired fibre orientations, the leaflet material response and valve performance were compared across different fibre reinforcement structures. For this, five models with different fibre configurations were investigated: bioinspired, all circumferential, all radial, isotropically dispersed, and no fibres.

The material models for all the fibre-reinforced structures were calibrated using a 2-layer MEW structure with a fibre spacing of 2 mm embedded in 0.3 mm of PDMS. The calibrated GOH parameters for this were: C_10_ = 0.078 MPa; k_1_ = 0.153 MPa; k_2_ = 0.109. Dispersion, κ, was set to zero for all fibre models except for the isotropic model, where it was set to 0.333. For the model without fibres, a Neo-Hookean material definition was used with C_10_ = 0.078.

### 2.4. Design of Experiments (DOE)

DOE was used to identify the best combinations of structural components in the leaflet. Three structural features with four discrete levels were investigated: number of MEW layers (2, 5, 8, 10), fibre spacing (0.5, 1, 1.5, 2 mm), and silicone thickness (0.2, 0.3, 0.4, 0.5 mm). These parameters were chosen based on feasible manufacturing ranges for layers and fibre spacing, and based on bioprosthetic valve leaflet thickness [36,37,38]. JMP Pro 18 (SAS Institute, Cary, NC, USA) was used to map the DOE runs; a response surface design was chosen with measured responses and the targets are shown in Table 1. Each response was weighted equally, except for average opening area and closed area, which were weighted 2×. These responses are representative of the limited features that can be assessed clinically, and, therefore, it is essential for valves to meet these requirements to be deemed functional in a clinical sense. Thirteen runs, 12 unique and 1 repeated combination, were identified to map this space fully; they are shown in Table 2.

### 2.5. Statistical Analysis

All statistical analyses related to the DOE were performed using JMP Pro 18, and *p* ≤ 0.05 was considered statistically significant.

## 3. Results

### 3.1. Fibre Orientation Analysis

**Figure 6:**
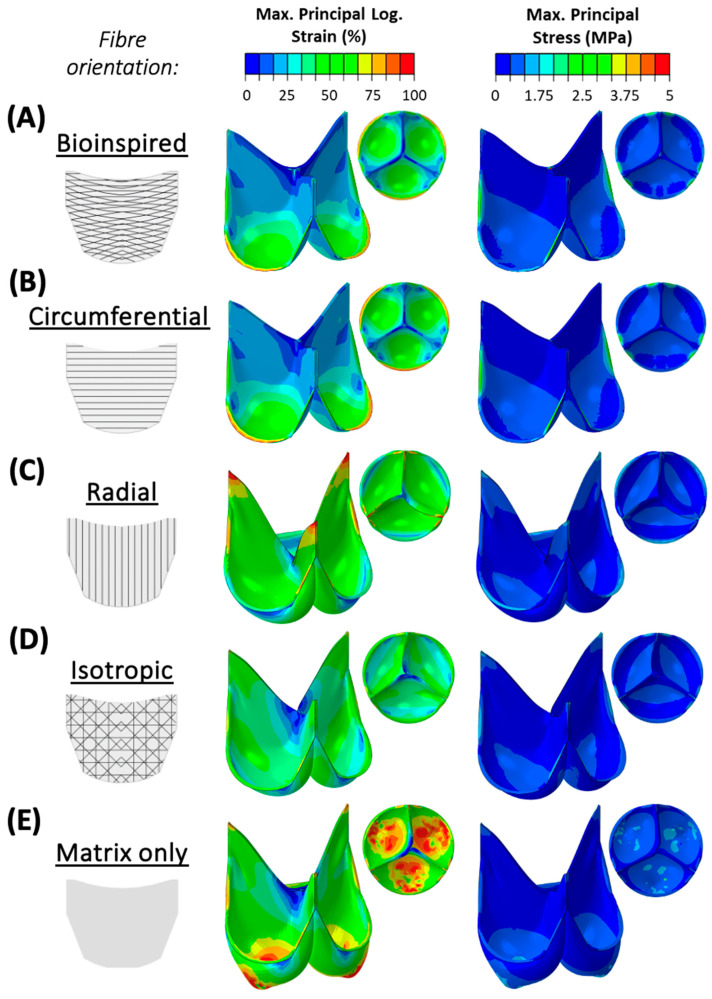
Strain and stress outputs from fibre orientation analysis showing (**A**) bioinspired orientation, (**B**) circumferential orientation, (**C**) radial orientation, (**D**) isotropic orientation, and (**E**) matrix only with no fibre reinforcement.

Maximum principal stress and strain plots of the five different fibre-reinforced leaflets are shown in Figure 6. Across all the valves, there were high levels of strain and low levels of stress. Both the bioinspired and circumferential orientations yielded valves that achieved full closure with minimal pinwheeling, whereas the other three orientations exhibited excessive leaflet deformation. Notably, the unreinforced, matrix-only leaflets reached a peak of 119.4% strain in the belly region, and the leaflets with radially aligned fibres reached 102.6% at the commissure edges. In the other three configurations, peak strains are far lower in magnitude and located at the bottom suture edge, with the isotropic configuration reaching 74.6% maximum strain, the circumferential reaching 69% maximum strain, and the bioinspired orientation reaching 66.4% maximum strain. Peak stresses in the leaflets occurred in the same regions. The three leaflets with the highest strain experienced the lowest peak stresses (radial: 1.25 MPa; isotropic: 1.34 MPa; matrix only: 1.57 MPa) while the bioinspired (2.34 MPa) and circumferential (2.53 MPa) orientations experienced higher peak stress.

**Figure 7:**
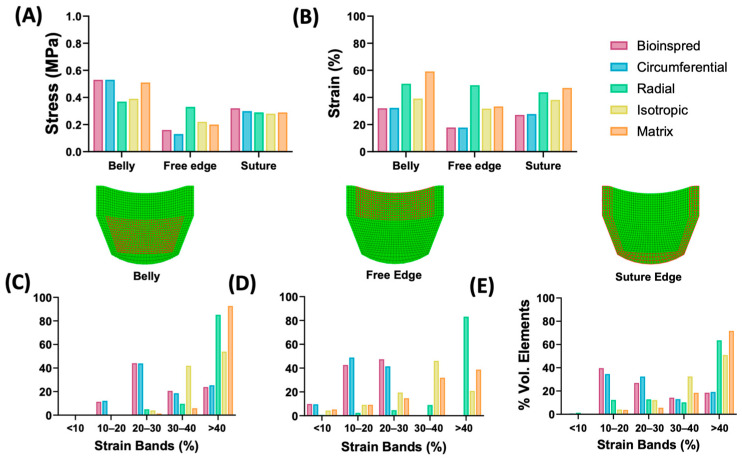
Stress and strain values extracted from models with differing fibre reinforcement structures at three different regions. (**A**) Average stress for each model in each region, (**B**) average strain for each model in each region, and volume percentage of elements in different strain bands for (**C**) belly, (**D**) free edge, and (**E**) suture edge.

Average stress and strain within the three leaflet regions are shown in Figure 7. Stress was highest in the belly region for all models, with the bioinspired, circumferential, and matrix-only models experiencing the highest average stress. In the free edge, the highest average stress was seen in the radial leaflets, while in the suture region, all of the leaflet configurations experienced similar levels of stress. Greater differences in reinforcement configurations are evident in strain levels, with both the bioinspired and circumferential models exhibiting lower strain than the other three models. In particular, the radial configuration and matrix-only models experienced greater strains across all three regions.

Strain in the leaflets was further investigated by organising the elements into percentage-volume bands of 10% strain; see Figure 7C–E. At the suture edge, the bioinspired and circumferential orientation models have most of the region experiencing less than 30% strain, whereas the other three configurations have most of the region above 40%. Similar trends are observed in the free-edge and belly regions, where the bioinspired and circumferential models have more elements in the lower-strain bands. In contrast, the other three models have large volumes in the higher-strain bands above 30% and 40%.

**Figure 8:**
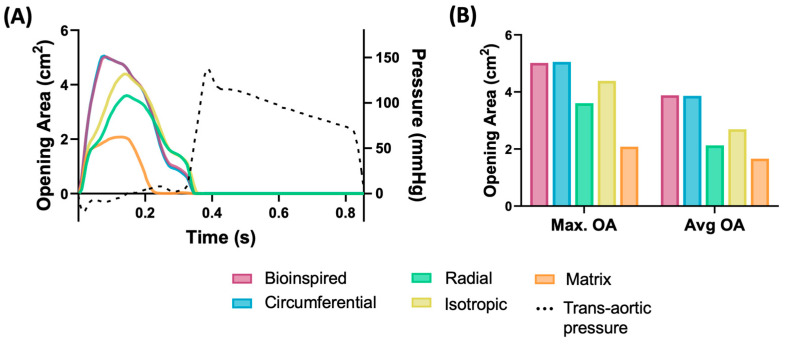
Opening area analysis for valve models with different fibre reinforcement structures. (**A**) Opening area for each model throughout the cycles, and (**B**) maximum and average opening area values for each configuration.

Opening areas throughout the cycle varied greatly between the models, with the bioinspired and circumferential configurations reaching the largest maximum OA and average OA; see Figure 8. The matrix-only leaflets had the worst performance, with the lowest maximum and average OAs, while the radial configuration performed slightly better.

### 3.2. Design of Experiments

#### 3.2.1. Valve Models

**Figure 9:**
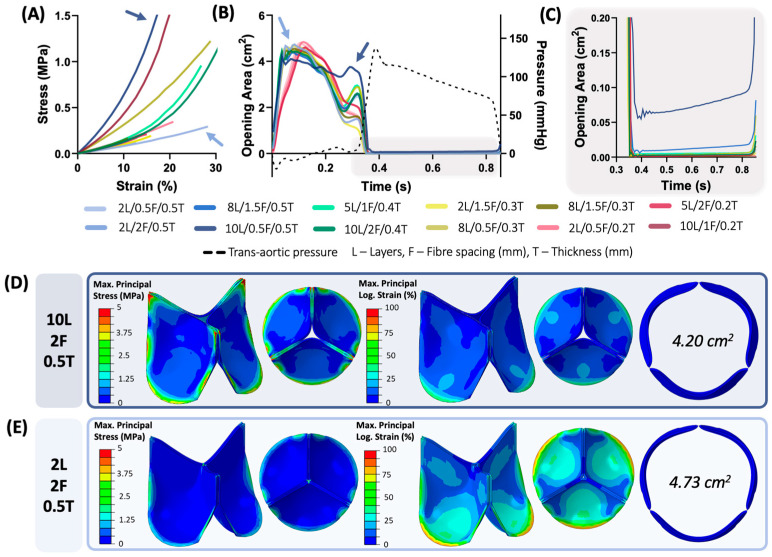
The results of all the models run in DOE analysis, showing (**A**) stress–strain curves from the uniaxial model, (**B**) opening areas throughout the pressure cycle, (**C**) a detailed view of the closed areas, and a detailed view of the valve models with (**D**) stiffest material response and (**E**) most compliant material response. The arrows in (**A**,**B**) indicate the response of the two valve examples given in (**D**,**E**).

Figure 9A shows the stress–strain response for each parameter combination and the corresponding valve opening area throughout the cycle. A wide range of stiffnesses was simulated across the 12 unique fibre/matrix structural combinations, resulting in a variation in OA responses. Looking at the stiffest combination (10 layers, 0.5 mm fibre spacing, and 0.5 mm thickness), there were high levels of stress at the suture edge with low strain across the leaflet; see Figure 9C. Conversely, the most compliant combinations (two layers, 2 mm fibre spacing, and 0.5 mm thickness) experienced low stress and high strain across the leaflet; see Figure 9D,E. Additionally, the more compliant material resulted in a larger maximum opening area (4.73 vs. 4.20 cm^2^) and achieved full closure, whereas the stiffer combination did not fully close, with an average closed area of 0.099 cm^2^.

#### 3.2.2. DOE Results

As shown in Table 3, the regression model provided a good fit for each response, with all R-squared values reaching over 0.95. Each factor, on its own, had numerous significant linear relationships with the responses, with the number of layers and fibre spacing significantly affecting the leaflet stress, strain, and maximum opening area. Fibre spacing also had a significant impact on the time to reach maximum opening area, and leaflet thickness had a strong significant impact on all responses except strain at the suture edge and the average closed opening area. There are fewer significant nonlinear relationships, all of which are less statistically significant than the linear fit, except for leaflet thickness and strain at the suture edge; this is the only nonlinear relationship with a higher *p*-value than the linear fit.Additional information on DOE fit can be found in Supplementary Materials, Table S1.

Combining the factors also yielded significant relationships in some cases, with the pairing of layer number and fibre spacing having a significant impact on stress in both regions of interest, as well as on the maximum and closed opening areas. The number of layers and thickness also had significant effects on the maximum and closed opening areas, as well as the time to reach maximum opening.

**Figure 10:**
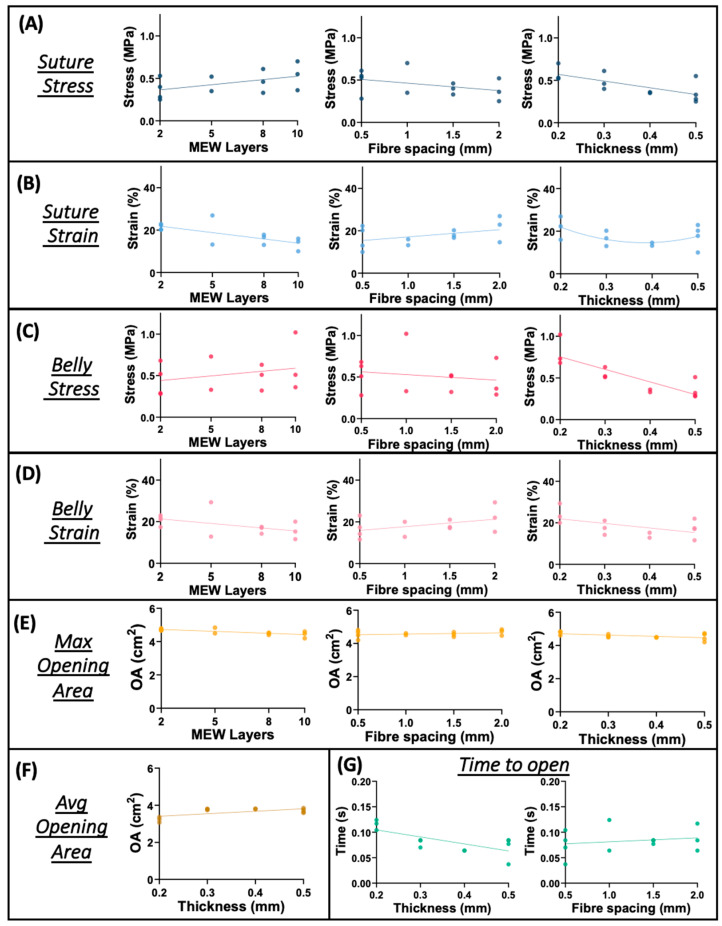
Scatter plots for every factor and response with a statistically significant relationship, including an indicative line of best fit. (**A**) Suture stress, (**B**) suture strain, (**C**) belly stress, (**D**) belly strain, (**E**) maximum opening area, (**F**) average opening area, and (**G**) time to open.

Figure 10 shows scatter plots for every factor and response pairing that had either a significant linear or nonlinear relationship. Stress in the two regions decreased with fewer layers, greater fibre spacing, and increased leaflet thickness (Figure 10A,C). A smaller fibre spacing and an increased number of layers reduced average strain (Figure 10B,D). A significant relationship was found between maximum opening area and all three factors, but the change in opening area for every factor was very small (Figure 10E). The average opening area was increased with increasing thickness, rising by 15.1% between 0.2 and 0.5 mm (Figure 10F). Notably, all recorded maximum and average opening areas were well above the 1.7 cm^2^ target. Finally, the time to maximum opening area decreased with greater leaflet thickness and smaller fibre spacing (Figure 10G).

**Figure 11:**
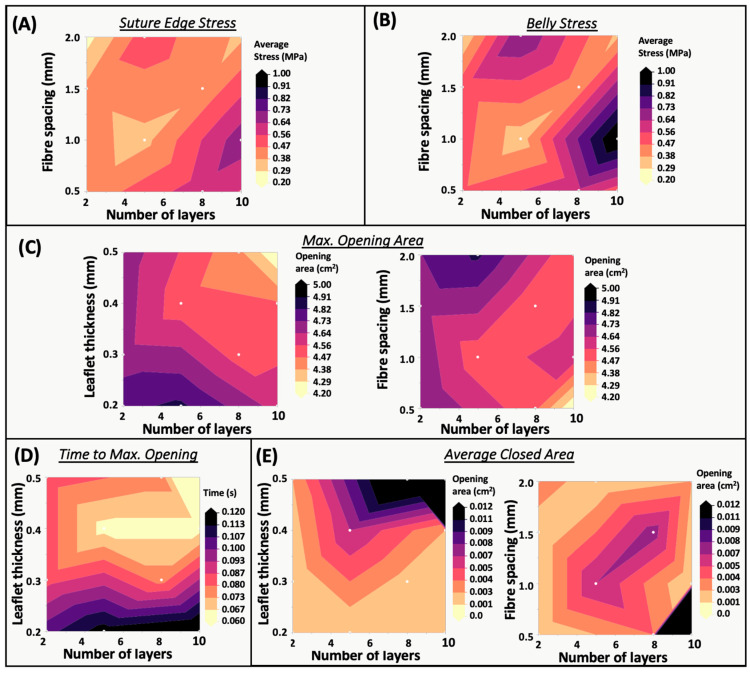
Heatmaps of all paired factors and responses with statistically significant relationships. (**A**) Suture edge stress, (**B**) belly stress, (**C**) maximum opening area, (**D**) time to maximum opening, (**E**) average closed area.

Pairings of factors and their results on responses are shown in Figure 11. These followed the same relationships described previously but showed an additional level of complexity in their response to these two factors. In both regions, stress increased with the number of layers and with smaller fibre spacing, although it was greater at 1 mm than at 0.5 or 1.5 mm (see Figure 11A,B). The maximum opening area increased with fewer layers, thinner thickness, and larger fibre spacing (see Figure 11C). The time to maximum opening decreased with increasing thickness; see Figure 11D. Closed area was the only response that showed a significant relationship with pairs of factors, with a smaller area for lower thickness and generally fewer layers. However, a large number of layers with a larger fibre spacing also improved leaflet closure; see Figure 11E. It should be noted, however, that all valves were effectively closed, and the recorded areas were small, measured in a small number of pixels.

#### 3.2.3. Most Desirable Combinations

**Figure 12:**
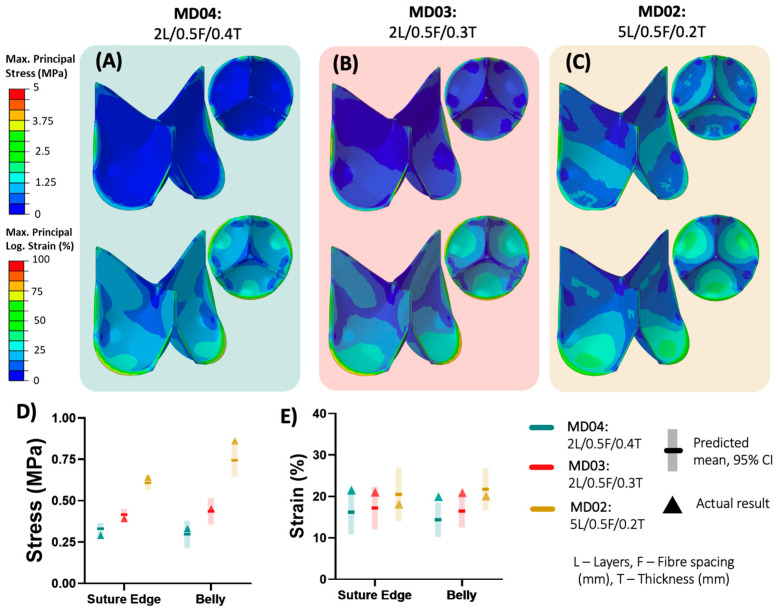
The stress and strain results of the three most desirable (MD) combinations: 2 layers, 0.5 mm fibre spacing, 0.4 mm thickness (MD04); 2 layers, 0.5 mm fibre spacing, 0.3 mm thickness (MD03); and 5 layers, 0.5 mm fibre spacing, 0.2 mm thickness (MD02). Visualisations of the stress and strain in leaflets for (**A**) MD04, (**B**) MD03, and (**C**) MD02, as well as results compared to DOE predictions for (**D**) stress in the suture edge and belly regions, and (**E**) strain in the suture edge and belly regions for all combinations.

From the results of all combinations, a “most desirable” combination of two layers, 0.5 mm fibre spacing, and 0.4 mm thickness was identified (named MD04; desirability: 0.78), as shown in Figure 12A. Due to the desire to maintain a low-profile device, the most desirable combinations for 0.3 mm and 0.2 mm thickness were also identified: two layers, 0.5 mm fibre spacing, 0.3 mm thickness (MD03, desirability: 0.65); and five layers, 0.5 mm fibre spacing, and 0.2 mm thickness (MD02, desirability: 0.1); see Figure 12B,C. These three combinations, not in the original DOE run, were created and run, and their results were compared to the software predictions. Average stresses in the leaflet suture edge and belly increased with decreasing thickness, while strain decreased in some regions and increased in others (see Figure 12D,E). These stress and strain measures were well predicted by the DOE, with each result falling within the corresponding confidence intervals for each response. Additionally, the DOE correctly predicted increased stress in these regions as leaflet thickness decreased. Strain levels in each of these regions also fall within or very close to the predicted intervals; however, MD02 experienced slightly lower average strain in these regions compared to MD04 and MD03 (MD02: 18.1% suture, 20.1% belly; MD03: 21% suture, 20.9% belly; MD04: 21.5% suture/19.9% belly), where the DOE predicted the strain would be slightly higher than the other two combinations (MD02: 20.5% suture/21.74% belly; MD03: 17.22% suture/16.49% belly; MD04: 16.21% suture/14.37% belly); see Figure 12E.

**Figure 13:**
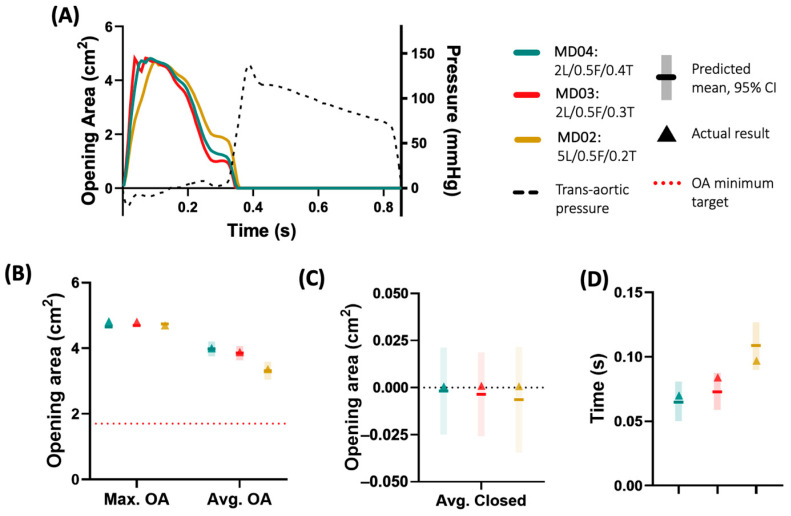
The opening area results for the three most desirable combinations, showing (**A**) opening areas versus pressure for a cycle, (**B**) maximum and average opening area results versus predictions, (**C**) average closed areas versus predictions, and (**D**) time to maximum opening versus predictions.

The opening areas across the cycle for each most-desirable combination are shown in Figure 13A. The maximum area reached was similar across valves (MD04: 4.82 cm^2^; MD03: 4.81 cm^2^; MD02: 4.7 cm^2^). In comparison, MD02 had a lower average opening area than the other two combinations (MD04: 4.01 cm^2^; MD03: 3.89 cm^2^; MD02: 3.67 cm^2^); see Figure 13B,C. Additionally, MD02 took slightly longer than the other two combinations to completely open (MD04: 0.07 s; MD03: 0.084 s; MD02: 0.097 s); see Figure 13D. All three valves reached full closure. These behaviours were predicted well, with all the results for maximum opening area, average opening area, average closed area, and time to maximum opening falling within the predicted intervals and very close to the mean (see Figure 13B–D).

## 4. Discussion

This work presents the creation of a finite element and Design of Experiments (FE-DOE)-based framework to aid the design of future bioinspired polymeric aortic heart valves. Using a fibre reinforcement structure manufactured with MEW, we developed a highly efficient methodology to obtain the mechanical behaviour of PEEK fibre-reinforced PDMS and simulate this material in a trileaflet valve model. After demonstrating the positive impact of implementing a bioinspired fibre structure in this leaflet geometry, this methodology was used in conjunction with DOE to determine the optimal structural combination of a PEEK-like fibre structure manufactured using MEW and embedded in PDMS.

The first step was to determine the stress/strain response of a PEEK fibre-reinforced PDMS in silico using a discrete fibre-reinforced dogbone model simulating a uniaxial tensile test. The response of this composite material was used to calibrate the relevant parameters of the GOH fibre-reinforced model, thereby significantly reducing the computational complexity and simulation time. Pressure was applied to the aortic and ventricular surfaces based on measurements from pulse duplicator testing of size large ACURATE neo2 valves. The pressure curves show minor differences from the commonly referenced Wiggers Diagram, most likely due to system setup and the resolution of the pressure measurements, but they align with other pressure–time curves given in the literature on bioprosthetic valves [38,39].

We showed the flexibility of this model and the impact of fibre orientations on material response and valve performance by simulating leaflets with varying reinforcement configurations. These results clearly showed the impact of different reinforcement structures on material strain and valve performance. These insights are corroborated by a study performed on similar leaflet geometry for porcine pericardium by Armfield et al. [38], where the authors showed greater leaflet displacement for radially aligned fibres than for circumferentially aligned fibres. Also, Serrani et al. [40] found a reduction in overall and maximum leaflet strain with an optimised fibre reinforcement structure compared to an isotropic orientation.

While the bioinspired and circumferential orientation models performed similarly, the bioinspired orientation reduced the peak strains and stresses at the suture edge, which could help extend material lifetime, as the material is most likely to fail here. Additionally, localised changes in the leaflet fibre orientations demonstrate the benefits of customised fibre reinforcement. A non-uniform fibre structure which supports different regions of the leaflet, as required by the local loading, enables bespoke tailoring to suit the particular leaflet’s needs. A bioinspired structure based on the native aortic valve leaflet was presented in this work, but there are geometrical differences (i.e., shape, thickness) between the native leaflets and those modelled in this study. It is likely that this particular reinforcement structure could be further optimised for this particular leaflet shape, but it is still providing a benefit in terms of leaflet response compared to a uniform circumferential arrangement of fibres. Therefore, this shows the potential of utilising a non-uniform reinforcement structure which varies across the leaflet and highlights the ability to tailor it to a specific leaflet shape.

For the analysis of these valve models, a robust, repeatable assessment framework was established to provide broad insights into leaflet performance and enable future design choices. Using DOE, we determined the impact of fibre spacing, number of layers, and overall thickness on leaflet and valve performance. A variety of responses were investigated, including both material behaviour and clinically relevant performance indicators of the opening area.

All of the models achieved good opening areas, far surpassing the 1.7 cm^2^ minimum for this size valve in both the maximum OA and average OA [35]. Additionally, all but the stiffest material combinations reached full valve closure. While the materials in each of these models may differ in their responses, these opening areas suggest that these stiffness ranges are good candidates for enabling effective aortic valves.

Greater differences in the leaflet structural combinations were seen in the stress and strain results, particularly in the belly region. To ensure a durable leaflet, the stress and strain in the material should be minimised. In addition, the models differed in the time required to reach maximum opening area, particularly for thinner leaflets, suggesting poorer valve efficiency. Using the DOE results, we can see that leaflet thickness had a significant impact on these responses, with greater thickness leading to reduced stress, strain, and time to reach maximum opening area. Fibre spacing and number of layers also had a significant impact on leaflet stress and strain, but in opposing ways: fewer layers and greater spacing increase strain while decreasing stress. A larger fibre spacing will also increase the time to maximum valve opening. Notably, no single factor affects valve performance and material response more than the others; all three have significant impacts on many responses. Meanwhile, almost all of the valves were fully closed, and none of the factors alone had a significant impact. While two combinations of factors (layer number and fibre spacing; layer number and thickness) yielded significant correlations with closed area, the actual values of the closure area are negligible.

The overall complexity of the factors’ contributions to the responses supports the use of this DOE approach. It balances the interactions among factors with the desired outcome for each factor (i.e., maximise or minimise) to provide a “most desirable” combination. We identified the most desirable structural combinations for the leaflets based on the input criteria outlined in Table 1. The most desirable combination identified was 0.4 mm thick, which is thicker than the porcine pericardium used in many self-expanding TAVR valves [36,38]. Due to the clinical need for a thinner leaflet to maintain a low-profile device when crimped, we also identified the best combinations at the two smaller thicknesses: 0.3 and 0.2 mm. These combinations were created, calibrated, and run in the dynamic valve model, and their results showed very close agreement with the DOE predictions. All the responses fell within the 95% predicted confidence interval bands relative to the mean, except for belly strain in the MD04 and MD03 models, whose actual values were 7% and 2% greater than the maximum predicted, respectively. Additionally, it was predicted that MD02 would experience higher strains than MD04 and MD03, but this was not the case. This is partly attributed to the smaller range of strain values observed across the most favourable fibre combinations, compared to the larger range of stress. Within the MD combinations, the maximum difference in strain was 17.2% in the suture region and 4.9% in the belly region, compared with stress differences of 76.6% and 89.1%, respectively. Consequently, small absolute errors in strain prediction have a greater effect on the apparent predictive accuracy of the DOE model. Nevertheless, the strain response retained predictive value, predicting values close to those experienced in the valve models. Therefore, the DOE model should be interpreted as a design-screening tool for identifying favourable fibre configurations, rather than as a fully quantitative predictor of local strain magnitude. Outside of this, the remaining responses were predicted very well. MD03 achieved results similar to, but overall slightly worse than, MD04 across all categories, suggesting that maintaining a low device profile with thinner leaflets necessitates a trade-off in the material’s mechanical response. Based on these results and the industrial need for thinner leaflets, the best combination in this dataset is likely MD03, as it is the thinnest option with the least compromise in material response.

A limitation of the present study is the use of the GOH constitutive model to represent the fibre-reinforced polymer composite. The GOH formulation was originally developed for anisotropic biological tissues and captures strain-stiffening behaviour associated with fibre reorientation and recruitment. However, it is not specifically formulated for synthetic fibre–polymer composites and may not fully capture the micromechanical behaviour of MEW reinforcement within an elastomeric matrix. As demonstrated in the Supplementary Materials, the model captures qualitative trends in stiffness variation as fibre orientation changes. Still, it does not consistently predict the quantitative magnitude of these effects. Consequently, the current simulations may not fully capture the extent of leaflet stiffening or softening associated with architectural variations. Nevertheless, the model provides a consistent and computationally efficient framework for exploring architecture-driven performance relationships within the proposed FE-DOE methodology which would not be possible using a discrete fibre model in a full valve. Future work will focus on improved material calibration using multiple deformation modes and on implementing alternative fibre-reinforced constitutive models, including HGO-C [41], bilinear fibre [42], and other formulations [31] tailored to composite materials, to enhance predictive accuracy.

Additional limitations of this study should also be acknowledged. Firstly, the complete stent frame was not included in the final simulation. It is known that the frame in the ACURATE neo2™ displaces under leaflet loading, which would impact the stress and strain in the leaflets [38], and potentially the opening and closing behaviour. Fluid flow was not included either, which is likely to impact the same behaviours. While future work should investigate the impact of frame deflection and fluid flow, these factors are unlikely to affect the DOE outcome, since all leaflet combinations are compared under the same conditions. Secondly, the fibres were modelled as a linear elastic PEEK-like material with no yield, with mechanical properties estimated from the literature [43,44]. While PEEK is a stiff polymer, it also has a low yield strain [32]. Future work should assess the impact of this and investigate the use of PCL, as it is the most widely used and accessible material for MEW.

On the DOE itself, some combinations of factors would not be physically possible to manufacture. For example, while a 10-layered structure made of 20 µm diameter fibres could theoretically be embedded within 0.2 mm of silicone, this would not be possible on the bench using MEW due to the high peaks formed when fibres overlap [45,46], resulting in a total thickness in these regions of greater than 0.2 mm. This would also pose manufacturing challenges for eight-layer structures at the same total leaflet thickness.

## 5. Conclusions

Overall, a FE-DOE framework has been established to enable the systematic, architecture-level optimisation of MEW fibre-reinforced polymer valve leaflets. By parameterising MEW-manufacturable fibre layouts and leaflet thickness within a fully in silico workflow, the study demonstrates how additive manufacturing design freedom can be quantitatively linked to valve-level performance and leaflet stress–strain response. This approach enables rapid identification of optimal structural configurations without the need for iterative fabrication, assembly, or experimental screening, thereby substantially accelerating the development cycle for fibre–polymer composite valve leaflets.

Although the present findings are specific to the material systems investigated, the methodology is inherently transferable to alternative polymer–fibre combinations and other MEW-enabled architectures. The framework provides a scalable digital design strategy for exploring high-dimensional additive manufacturing parameter spaces, including fibre orientation, spacing, layering strategy, thickness, fibre diameter, matrix stiffness, and potentially even process-dependent variables associated with MEW. By integrating finite element modelling with structured design exploration, the study establishes an optimisation pathway to guide targeted in vitro validation while minimising the experimental burden.

## Figures and Tables

**Table 1:** Responses investigated using DOE, with objective and target for each, if applicable.

Response	Objective	Target	Weight
Average stress (belly and suture edge)	Minimise	--	1×
Average strain (belly and suture edge)	Minimise	--	1×
Maximum opening area	Maximise	Minimum: 1.7 cm^2^	1×
Average opening area	Maximise	Minimum: 1.7 cm^2^	2×
Average closed area	Minimise	0 cm^2^	2×
Time to opening	Minimise	--	1×

**Table 2:** Embedded MEW structure combination runs as indicated by DOE.

Number of Layers—L	Fibre Spacing—F (mm)	Thickness—T (mm)
2	0.5	0.5
2	0.5	0.2
2	1.5	0.3
2	2	0.5
5	1	0.4
5	1	0.4
5	2	0.2
8	0.5	0.3
8	1.5	0.5
8	1.5	0.3
10	0.5	0.5
10	1	0.2
10	2	0.4

**Table 3:** The relationships between all input factors and the resulting response. The red numbers indicate a significant *p*-value less than 0.05, and the yellow numbers indicate a *p*-value less than 0.01. L: layer number; F: fibre spacing; T: thickness. ns means non-significant.

Response	Fit R^2^	Factors								
		L	L^2^	F	F^2^	T	T^2^	L×F	L×T	F×T
Suture edge stress	0.99	0.0005	ns	0.0009	ns	0.0001	0.0322	0.0021	ns	ns
Suture edge strain	0.96	0.0175	ns	0.0224	ns	ns	0.0369	ns	ns	ns
Belly stress	0.99	0.0046	0.0323	0.0326	ns	0.0002	0.0085	0.0137	ns	ns
Belly strain	0.98	0.0224	ns	0.0099	ns	0.0059	0.0159	ns	ns	ns
Max OA	0.99	<0.0001	ns	0.0002	0.0018	<0.0001	0.0008	0.0016	0.0003	ns
Avg. closed OA	0.96	ns	ns	ns	ns	ns	ns	0.0407	0.0313	ns
Avg. positive OA	0.98	ns	ns	ns	ns	0.0047	0.0057	ns	ns	ns
Time to max OA	0.99	ns	ns	0.0439	ns	0.0016	0.0056	ns	0.0158	ns

## Data Availability

Data will be made available on request.

## Supplementary Materials

The following supporting information can be downloaded at https://www.mdpi.com/article/10.3390/biomimetics11060421/s1, Figure S1: Comparison of PDMS mechanical response to a calibrated first-order Ogden model; Figure S2: Mesh sensitivity analysis of dogbone model; Figure S3: Mesh size sensitivity analysis; Figure S4: Detailed images of leaflet and frame boundary conditions; Figure S5: Opening area of porcine pericardium model compared to resultant aortic pressure; Figure S6: A schematic representation of the MEW fibre reinforcement architectures used in the study, showing fibre orientations of 15°, 45°, and 75° relative to the loading direction; Figure S7: A comparison of predicted true stress–strain behaviour for a MEW-reinforced polymer composite dogbone specimen with five fibre layers, 1 mm pore spacing, and 0.4 mm total thickness. The results are shown for discrete fibre modelling and a homogenised GOH continuum representation for fibre orientations of 15°, 45°, and 75° relative to the loading direction; Figure S8: A comparison of predicted true stress–strain behaviour for a MEW-reinforced polymer composite dogbone specimen with eight fibre layers, 1.5 mm pore spacing, and 0.3 mm total thickness; Table S1: R^2^ and DOE-adjusted R^2^ values for each DOE response.

## Abbreviations

The following abbreviations are used in this manuscript:

DOE	Design of Experiments
EOA	Effective opening area
FE	Finite element
GOH	Gasser–Ogden–Holzapfel (model)
MD	Most desirable
MEW	Melt electrowriting
OA	Opening area
PDMS	Polydimethylsiloxane
PEEK	Polyether ether keytone
TAVI	Transcatheter valve implantation
