# Supplementary material for: Optimisation of Bioinspired Fibre Architectures for 3D-Printed Polymer Heart Valves via Melt Electrowriting (MEW) Using FE Modelling and Design of Experiments (FE-DOE)
Source: Biomimetics (Basel). 2026 Jun 13;11(6):421. doi: 10.3390/biomimetics11060421 (PMC13297344; doi:10.3390/biomimetics11060421)
Supplement: Supplementary file 1 [file biomimetics-11-00421-s001.zip › biomimetics-4346954-supplementary.pdf]

Supplementary Material

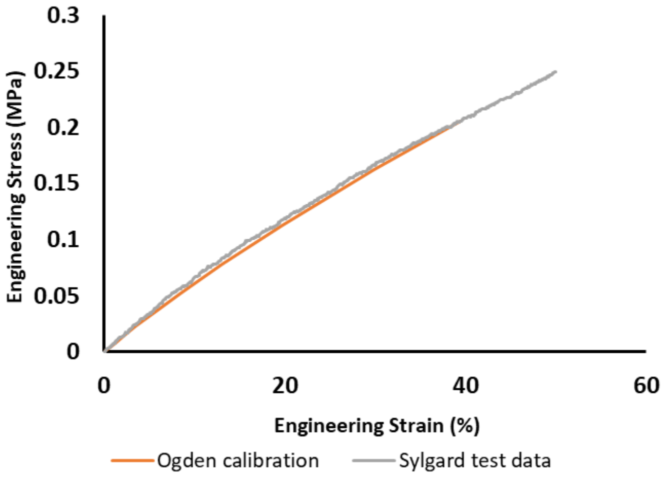

Figure S1 *Comparison of PDMS mechanical response to a calibrated first-order Ogden model (μ1= 0.204 MPa, α1= 2.504, and D=0).*

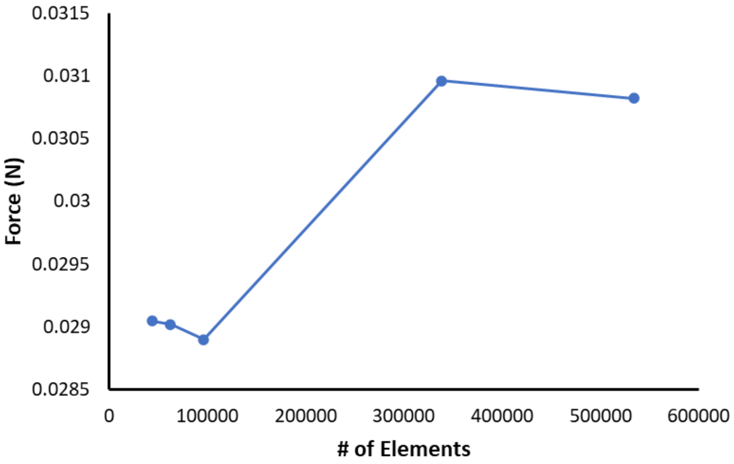

Figure S2 *Mesh sensitivity analysis of dogbone model.*

The reaction force at 20% strain for a variety of element numbers. Convergence was achieved by 35,000 elements, which corresponded to a seed size of 0.05 mm for the fibres and 0.2 mm for the matrix.

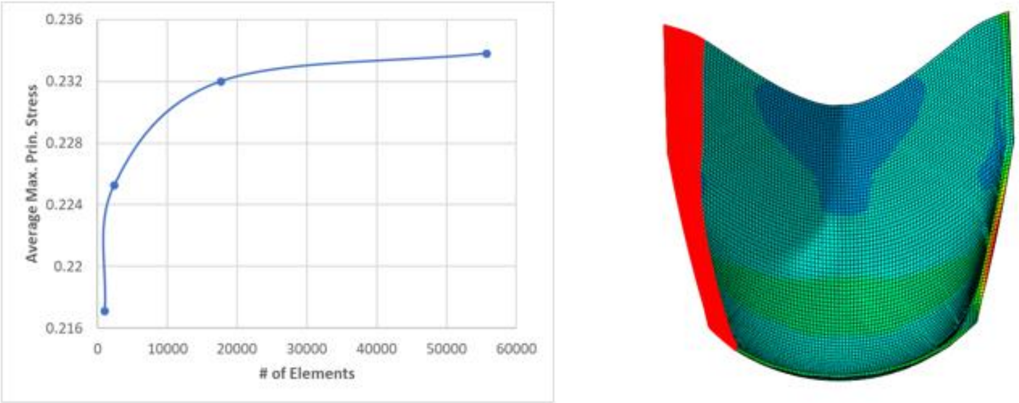

Figure S3 *Mesh size sensitivity analysis.*

The average maximum principal stress along the suture edge was analysed for increasing mesh densities and convergence was achieved at around 20,000 elements.

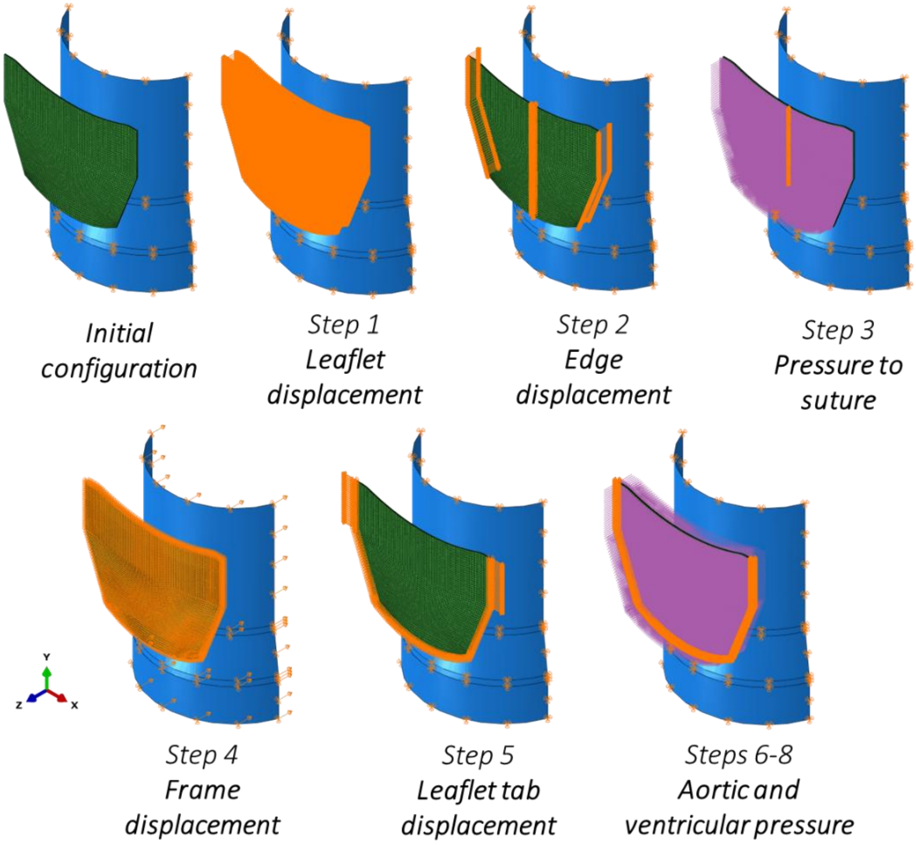

Figure S4 *Detailed images of leaflet and frame boundary conditions.*

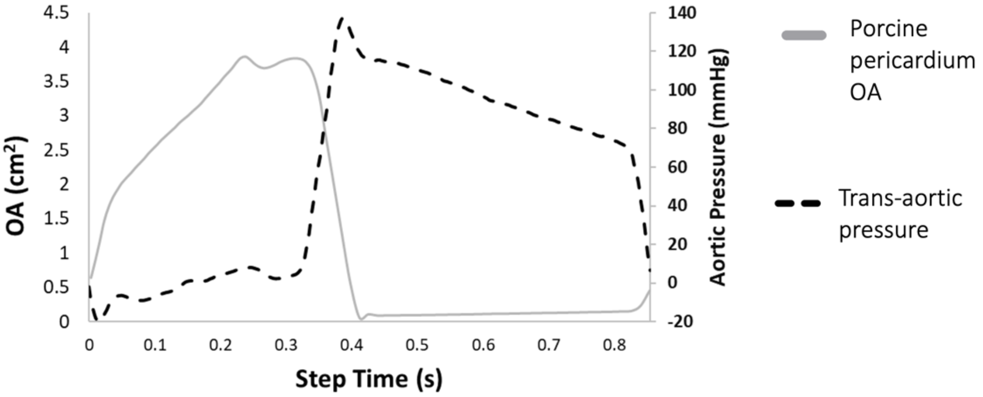

Figure S5 *Opening area of porcine pericardium model compared to resultant aortic pressure.*

The material properties of highly dispersed porcine pericardium were applied to the trileaflet valve model, and the average OA was compared to expected EOA ranges provided by Boston Scientific to validate the comparability of these outputs. The valve model accurately predicted the measured EOA values, with an average OA of 2.14 cm^2, within the expected range of 2.0–3.0 cm^2.

To evaluate the suitability of the Gasser–Ogden–Holzapfel (GOH) constitutive model for representing melt electrowritten (MEW) fibre-reinforced polymer architectures, uniaxial tensile simulations were performed using two modelling approaches. In the first approach ("Discrete"), the reinforcing fibres were explicitly represented within the elastomeric matrix using explicitly modelled fibre elements arranged in the architecture corresponding to the printed MEW layout (refer to Figure 1). In the second approach ("GOH"), the composite was represented as a homogeneous continuum using the GOH anisotropic material model, with fibre directions aligned with the corresponding reinforcement architecture.

Dogbone specimens were modelled in Abaqus and subjected to uniaxial tension. Fibre orientations of 15°, 45°, and 75° relative to the loading direction (Figure S6) were considered, representing elongated diamond architectures aligned with the loading direction, square architectures, and transverse diamond architectures, respectively.

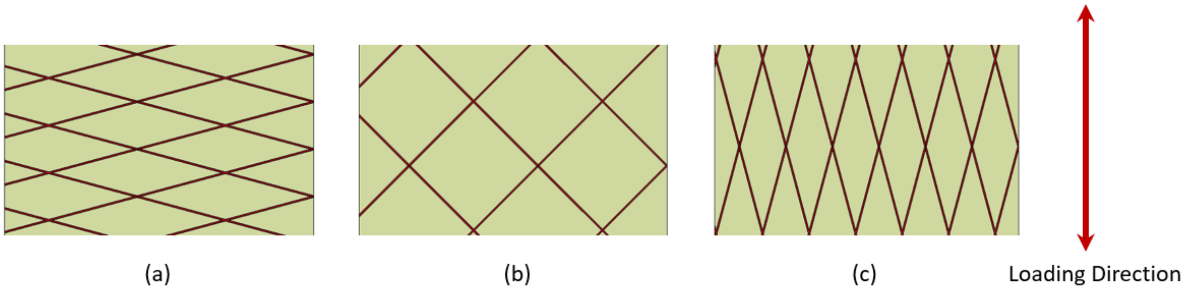

Figure S6 *A schematic representation of the MEW fibre reinforcement architectures used in the study, showing fibre orientations of 15°, 45°, and 75° relative to the loading direction.*

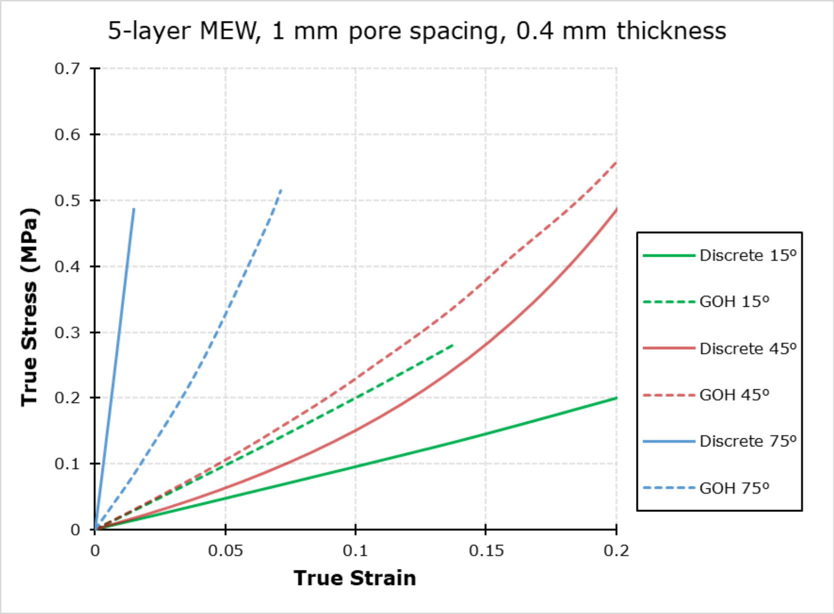

Figure S7 *A comparison of predicted true stress–strain behaviour for a MEW-reinforced polymer composite dogbone specimen with five fibre layers, 1 mm pore spacing, and 0.4 mm total thickness. The results are shown for discrete fibre modelling and a homogenised GOH continuum representation for fibre orientations of 15°, 45°, and 75° relative to the loading direction.*

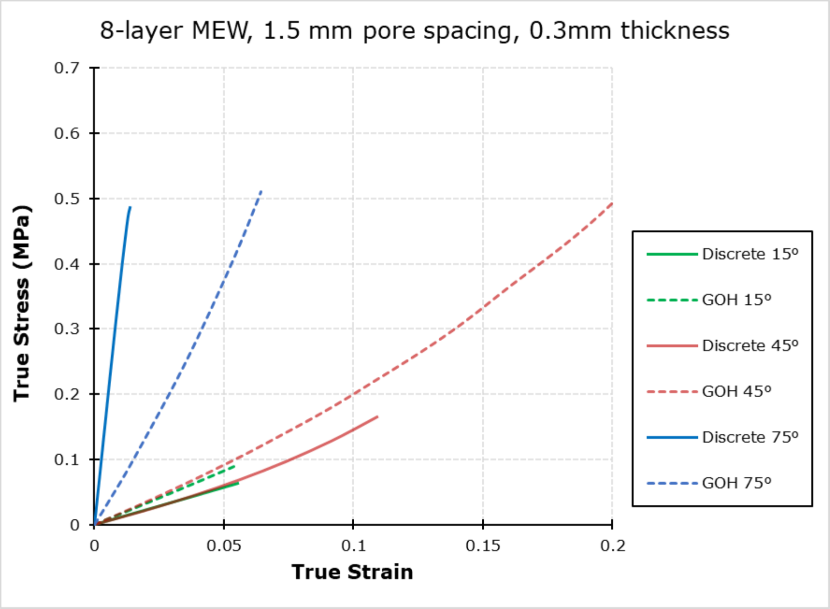

Figure S8 *A comparison of predicted true stress–strain behaviour for a MEW-reinforced polymer composite dogbone specimen with eight fibre layers, 1.5 mm pore spacing, and 0.3 mm total thickness.*

Figures S7 and S8 compare the predicted stress–strain response of the discrete fibre models and the homogenised GOH representation for two representative MEW reinforcement configurations. In both cases, the GOH formulation captures the qualitative influence of fibre orientation on

structural stiffness. Architectures with fibres more closely aligned with the loading direction exhibit increased stiffness, whereas transverse architectures produce a more compliant response. However, the continuum GOH model does not consistently reproduce the quantitative magnitude of these effects relative to the discrete fibre representation.

These results indicate that, while the GOH formulation provides a computationally efficient approximation capable of capturing orientation-dependent trends, it does not fully resolve the micromechanical response of the discrete MEW fibre architecture. Nevertheless, it provides a suitable representation for exploring architecture–performance relationships within the FE-DOE framework employed in this study.

Table S1 *R^2 and DOE-adjusted R^2 values for each DOE response.*

Response	Fit R^2	Adjusted R^2
Suture edge stress	0.99	0.99
Suture edge strain	0.96	0.85
Belly stress	0.99	0.98
Belly strain	0.98	0.91
Max OA	0.99	0.99
Avg. closed OA	0.96	0.93
Avg. positive OA	0.98	0.90
Time to max OA	0.99	0.95
